# The relationship between transgenerational acquired resistance and global DNA methylation in Arabidopsis

**DOI:** 10.1038/s41598-018-32448-5

**Published:** 2018-10-03

**Authors:** Joost H. M. Stassen, Ana López, Ritushree Jain, David Pascual-Pardo, Estrella Luna, Lisa M. Smith, Jurriaan Ton

**Affiliations:** 1 Department of Animal and Plant Sciences, Faculty of Science and P3 Centre for Translational Plant Science, Western Bank, University of Sheffield, Sheffield, S10 2TN United Kingdom; 2 Present Address: Department of Plant Molecular Genetics, Spanish National Centre for Biotechnology, CSIC. Campus de Cantoblanco, C/ Darwin 3, Madrid, 28049 Spain; 3 Present Address: AgriBio, ARC centre of Excellence in Plant Energy Biology, School of Life Science, La Trobe University, 5 Ring Road, Bundoora, VIC 3083 Australia; 4 Present Address: School of Biosciences, University of Birmingham, Edgbaston, B15 2TT United Kingdom

## Abstract

Progeny of heavily diseased plants develop transgenerational acquired resistance (TAR). In Arabidopsis, TAR can be transmitted over one stress-free generation. Although DNA methylation has been implicated in the regulation of TAR, the relationship between TAR and global DNA methylation remains unknown. Here, we characterised the methylome of TAR-expressing Arabidopsis at different generations after disease exposure. Global clustering of cytosine methylation revealed TAR-related patterns in the F3 generation, but not in the F1 generation. The majority of differentially methylated positions (DMPs) occurred at CG context in gene bodies. TAR in F3 progeny after one initial generation of disease, followed by two stress-free generations, was lower than TAR in F3 progeny after three successive generations of disease. This difference in TAR effectiveness was proportional to the intensity of differential methylation at a sub-set of cytosine positions. Comparison of TAR-related DMPs with previously characterised cytosine methylation in mutation accumulation lines revealed that ancestral disease stress preferentially acts on methylation-labile cytosine positions, but also extends to methylation-stable positions. Thus, the TAR-related impact of ancestral disease extends beyond stochastic variation in DNA methylation. Our study has shown that the Arabidopsis epigenome responds globally to disease in previous generations and we discuss its contribution to TAR.

## Introduction

Due to their lack of mobility, plants rely heavily on phenotypic plasticity to adapt to environmental stress, including pests and diseases. Although the plant innate immune system provides full protection against the majority of potentially harmful microbes^1^, the level of disease pressure in natural environments can vary over time. Accordingly, plants have evolved the ability to adjust the sensitivity of their immune system in accordance to previous exposures to biotic stress. For instance, plants respond more effectively to pathogen attack after previous exposure to disease or other defence-eliciting signals^2^. This heightened immune responsiveness, or ‘defence priming’, results in an increased level of basal resistance, which is commonly referred to as ‘induced resistance’ or ‘acquired resistance’. Despite the benefit of increased protection, acquired resistance is associated with costs, such as reductions in plant growth and seed set^3^. Furthermore, acquired resistance against biotrophic pathogens, which relies on salicylic acid (SA)-dependent signalling, can reduce defence against necrotrophic pathogens and herbivores, which is controlled by jasmonic acid (JA)-dependent signalling^4^. Thus, acquired resistance gears the plant to respond more effectively against attackers with a similar infection strategy^5,6^, but this adaptation can be at the expense of resistance against other attackers^7^.

Acquired resistance can be effective over various time-scales, ranging from days to the lifetime of the individual. Furthermore, some acquired resistance responses can be transmitted to following generations^8–10^. Because this transgenerational acquired resistance (TAR) can occur in isogenic plant populations after only one generation of biotic stress, epigenetic mechanisms were proposed to underlie this phenomenon^11^. Indeed, TAR has recently been linked to epigenetic changes, including DNA methylation and histone modifications^8,12,13^. Furthermore, stress-inducible epigenetic modifications can develop within one generation and are often reversible, enabling fine-tuning of adaptive phenotypes in a changeable environment. Accordingly, epigenetic mechanisms offer an ecologically plausible mechanism of TAR, allowing plants to transmit defence traits to their progeny, without irreversibly fixing the resistance as a genetic trait, along with the associated costs^11,14^.

In order to provide an ecological benefit, TAR should be stress-inducible, inheritable and reversible in the absence of stress. Various epigenetic mechanisms, including histone modifications and DNA methylation, have been described as being stress-inducible, reversible and capable of modifying resistance phenotypes^8,9,12,13,15–20^. However, only DNA methylation of cytosines is known to be transmitted faithfully through meiosis and multiple generations^21–23^. DNA methylation and histone modifications are closely interrelated, which is evidenced by studies showing that mutations affecting the one modification often also affect the other^24–26^. This link has also been reported for TAR in Arabidopsis. For instance, mutants in RNA-directed DNA methylation (RdDM) show histone modifications at selected defence gene promoters that also occur in TAR-expressing progeny from disease-exposed wild-type plants^8,17^. Further support for a role of DNA methylation in TAR comes from the finding that disease exposure induces widespread within-generation changes in cytosine methylation^15^, which can alter the responsiveness and expression of defence genes^19^. Moreover, mutants in DNA (de)methylation machinery are affected in TAR^13,18^, even though these mutants are not impaired in the expression of within-generation systemic acquired resistance (SAR)^13,18^. Hence, mutations affecting DNA (de)methylation do not directly impair plant defence signalling, but affect the transmission and/or establishment of TAR.

DNA methylation in plants occurs at three sequence contexts: CG, CHG and CHH, where H is an A, T or C nucleotide. Disease stress has been reported to induce genome-wide changes in DNA methylation at every sequence context^15^. The maintenance and stability of these types of DNA methylation over cell division is controlled by different mechanisms. Methylation at CHH sites is relatively unstable, because it requires constant production of siRNAs and activity of the RdDM pathway to ensure on-going *de novo* methylation^27^. Conversely, CHG and CG methylation can be maintained independently of small RNAs, because the methylated cytosines can be copied directly from parent to daughter strand. In the case of CHG methylation, this process is mediated by a feedback loop that involves the histone methyltransferase KYP and the DNA methyltransferase CMT3^24^. CG methylation is maintained by MET1 and is considered to be the most stable DNA methylation context over cell division and meiosis^27^. This explains why the majority of previously reported heritable epi-mutations occur at CG sites^28–31^. Interestingly, salt stress has been reported to increase changes in heritable CG DNA methylation in genic regions of Arabidopsis^31^, suggesting that environmental stress can accelerate the occurrence of epi-mutations in coding gene sequences.

Despite evidence for a role of DNA methylation in TAR, the global methylome of TAR-expressing plants has never been characterised. Furthermore, whilst TAR has been reported to persist over one stress-free generation^8^, the transgenerational stability of disease-induced changes in DNA methylation remains unknown. Here, we have addressed these questions by determining global DNA methylation patterns in TAR-expressing plants at different generations after initial disease exposure.

## Results

### DNA methylation patterns and disease resistance in F1 progeny of *Pseudomonas syringae*-stressed plants

**Figure 1:**
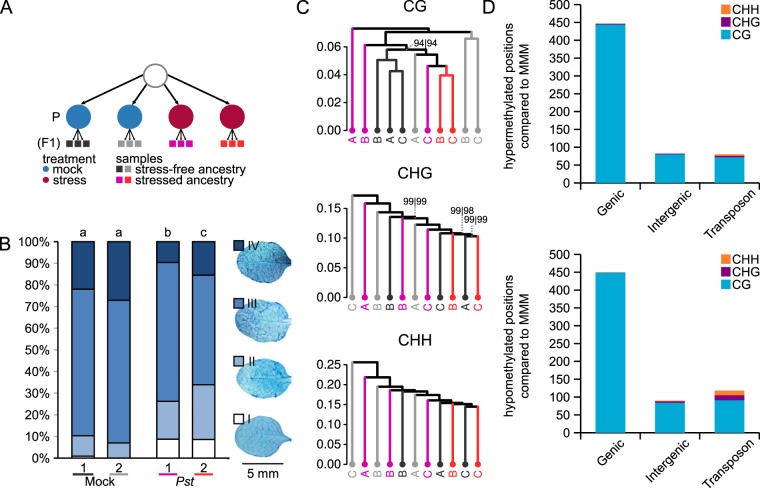
Global patterns of DNA methylation in F1 progenies expressing transgenerational acquired resistance (TAR) after one generation of disease stress. (**A**) Ancestry of the analysed F1 progenies. Shown are replicate lines that for one generation had been mock-treated (blue circles) or exposed to disease stress by *Pseudomonas syringae* pv. *tomato* (*Pst*; red circles). Bars of the same colour at the bottom of the scheme indicate replicate populations within each line (n = 10 plants) used for bisulfite-sequencing analysis. (**B**) Quantification of TAR in the four different F1 lines. Shown are relative abundances of leaves from 3 week-old plants over four distinct *Hyaloperonospora arabidopsidis* (*Hpa*) colonisation classes, quantifying selective pathogenesis-related marks during interaction with the oomycete. Class I: no hyphal colonisation; class II: hyphal colonisation but no sporulation; class III: hyphal colonisation with formation of conidiophores; class IV: extensive hyphal colonisation with conidiophores and oospores. Darker colours indicate increasing degrees of *Hpa* colonisation. Different letters above the bars indicate statistical differences (Fisher’s exact, all-versus-all, FDR-adjusted; *p* ≤ 0.05). (**C**) Hierarchical clustering (Pearson correlation, Ward) of cytosine methylation profiles at CG, CHG and CHH sequence contexts. Lines are colour-coded to match the line annotations in panel A. Letters indicate the three replicate population samples within a line. Numbers at edges indicate AU (approximately unbiased) and BP (bootstrap probability) *p*-values (%), respectively. Confidence values are only shown for edges where AU or BP *p*-values are < 100. (**D**) Number of differentially methylated positions (DMPs) mapping to genomic features. DMPs were defined as population differences that were statistically significant between all replicate samples from both mock-inoculated lines and all replicate samples from both *Pst-*treated lines (Logistic regression; *q*-value < 0.05). Features are defined as transposon, gene (including 5′ UTR, intron, exon and 3′ UTR, where defined) and intergenic (TAIR 10). Top panel: hyper-methylated DMPs; Bottom panel: hypo-methylated DMPs.

To study the relationship between DNA methylation and TAR, we first analysed patterns of global DNA methylation in TAR-expressing plants at the first (F1) generation after disease exposure. Background variation due to potential carry-over effects from stress in previous generations were minimised by harvesting seed from a single individual plant that had been propagated under stress-free conditions over at least three generations. From this seed stock, control and TAR-expressing lines were generated by repeated inoculations with either the mock solution (10 mM MgSO4), or the solution containing *Pseudomonas syringae* pv. *tomato* DC3000 (*Pst*), respectively. Repetitive inoculations with *Pst* have previously been shown to elicit TAR in Arabidopsis through priming of SA-dependent defences^8^. To confirm TAR in the current experiment, F1 progenies from two mock-inoculated plants and two *Pst*-inoculated plants were examined for resistance against the biotrophic pathogen *Hyaloperonospora arabidopsidis* (*Hpa*; Fig. 1A), which is taxonomically unrelated to *Pst*, but similarly resisted by SA-dependent defences^32,33^. As observed previously^8^, progeny from the two *Pst*-inoculated plants developed higher levels of resistance to *Hpa* than progeny from the two mock-inoculated plants (Fig. 1B).

To assess the impact of TAR on global DNA methylation, triplicate samples from progeny of all four plants were subjected to whole-genome bisulfite sequencing. Each replicate consisted of a pool of four similarly aged leaves from 10 healthy plants. Hence, one sample represents the average cytosine methylation within a population of 10 plants. A single replicate of one of the *Pst*-treated lines was discarded from further analyses because it’s sequencing coverage and methylome pattern differed significantly from all other samples. Cluster analysis of cytosine methylation (Pearson correlation, Ward) failed to group replicate samples within each progeny line, nor did it group progenies by ancestral treatment. This lack of global methylation patterning was apparent at all sequence contexts (Fig. 1C). Although principle component analysis (PCA) of CG methylation showed weak clustering of replicate samples within each line, it failed to separate mock-treated lines from *Pst*-treated lines (Fig. S1A). Furthermore, PCA of CHG and CHH failed to reveal TAR-related clustering of samples (Fig. S1A). Together, this indicates that a potential impact of paternal disease stress was either absent, or masked by spontaneously occurring variation in cytosine methylation.

To further examine TAR-related changes in DNA methylation, we selected for differentially methylated positions (DMPs) that show a statistically significant difference in average cytosine methylation between all samples from the *Pst*-inoculated lines and all samples from the mock-inoculated lines, using a maximum false discovery rate of 5% and over-dispersion correction. A total of 1,267 differentially methylated positions (DMPs) were detected with approximately similar numbers of hyper- and hypo-methylated positions (Fig. 1D). Almost all DMPs occurred in CG context (96.3%). Furthermore, the majority of these DMPs occurred at genic sequences.

### DNA methylation patterns and disease resistance in F3 progeny of *Pst*-stressed plants

**Figure 2:**
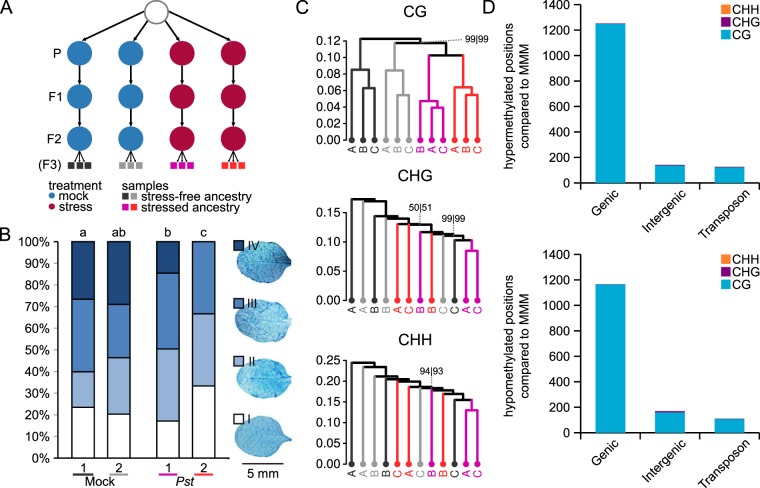
Global DNA methylation in F3 progenies expressing TAR after three successive generations of disease stress. (**A**) Ancestry of the F3 progenies analysed (first F3 experiment). Shown are replicate lines that for three successive generations had been mock-treated (blue circles) or exposed to disease stress by *Pst* (red circles). Bars of the same colour at the bottom of the scheme indicate replicate populations within each line (n = 10 plants) used for bisulfite-sequencing analysis. (**B**) Quantification of TAR in the four different F3 lines. Shown are relative abundances of leaves from 3 week-old plants over four distinct *Hpa* colonisation classes. For details, see Fig. 1B. Different letters above the bars indicate statistical differences (Fisher’s exact, all-versus-all, FDR-adjusted; *p* ≤ 0.05). (**C**) Hierarchical clustering (Pearson correlation, Ward) of cytosine methylation profiles at CG, CHG and CHH sequence contexts. Lines are colour-coded to match the line annotations in panel A. Identical letters indicate replicate population samples within a line. Numbers at edges indicate AU (approximately unbiased) and BP (bootstrap probability) *p*-values (%), respectively. Confidence values are only shown for edges where AU or BP *p*-values are < 100. (**D**) Number of DMPs mapping to genomic features. For details, see Fig. 1D. Top panel: hyper-methylated DMPs; Bottom panel: hypo-methylated DMPs.

The transgenerational effects of salt stress on DNA methylation have been reported to become more pronounced after multiple generations of stress^34^. Taking this into account, a new set of independent lines were created that spanned three generations of disease exposure (Fig. 2A). Progenies of this F3 experiment were tested for TAR against *Hpa* and subjected to whole-genome bisulfite-sequencing analysis. To shorten the generation time in this experiment, plants were grown under long day conditions, resulting in earlier flowering times. As a consequence, plants could only be inoculated three times per generation, resulting in generally lower disease levels than previous experiments, where plants were inoculated five to six times^8^. Accordingly, the strength of the TAR response between both *Pst*-exposed lines was more variable. Whereas F3 progeny from the first *Pst*-exposed line showed a relatively weak TAR response, which was statistically significant compared to progeny from one mock-treated line only, F3 progeny from the second *Pst*-exposed line showed a stronger TAR response, which was statistically significant compared to F3 progeny from both mock-treated lines (Fig. 2B).

To assess global patterns of DNA methylation, triplicate samples per F3 progeny, each consisting of a pool of four similarly aged leaves from 10 healthy plants, were collected for whole-genome bisulfite sequencing. Global analysis of CG methylation patterns by either Pearson correlation or PCA revealed that samples within progeny lines were highly similar (Figs 2C and S1B). Furthermore, in contrast to F1 progeny, F3 lines exposed to *Pst* treatment grouped together for CG methylation, suggesting a global impact of *Pst* stress in previous generations (Figs 2C and S1B). Interestingly, these global clustering patterns were not evident for CHG and CHH methylation (Figs 2C and S1B). Of the 2,941 DMPs at CG context, 1,509 were hyper-methylated, whereas 1,432 were hypo-methylated (Figs 2D and S2; Table S2). As with our F1 lines, differential methylation between the control and stress-exposed F3 lines predominantly occurred at CG context (99.1%) and genic sequences.

### The endurance of disease stress in previous generations is proportional to the level of TAR and differential CG methylation in F3 progeny

**Figure 3:**
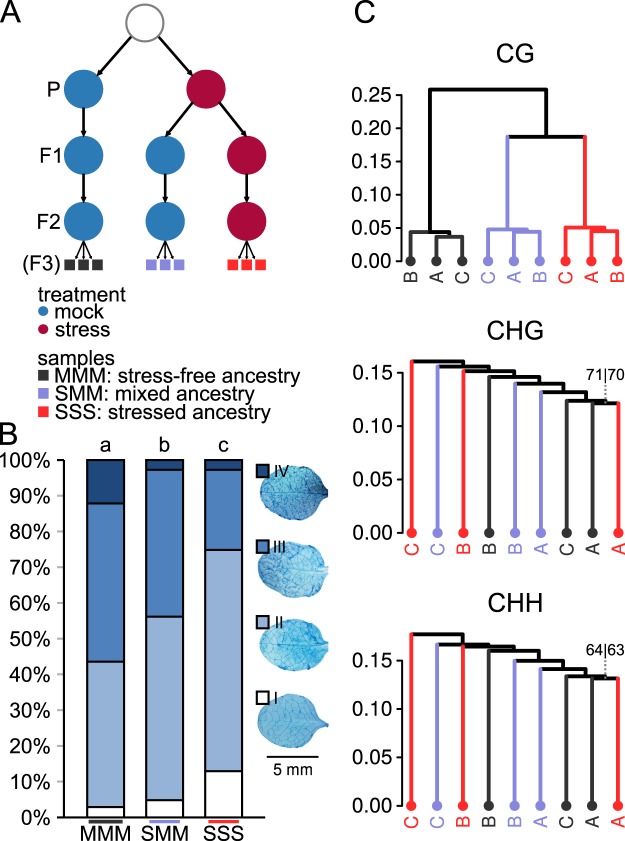
Global patterns of DNA methylation in F3 progenies expressing TAR after one initial generation or three successive generations of disease stress. (**A**) Ancestry of the F3 progenies analysed (second F3 experiment). Shown are lines that been mock-treated (blue circles) for three successive generations (MMM lines), exposed to one initial generation of *Pst* stress (red circles) followed by two mock-treated generations (SMM), or exposed to three successive generations of *Pst* stress (SSS). Blocks of the same colour the bottom of the scheme indicate replicate populations (n = 10 plants; grey squares: MMM, orange squares: SMM, red squares: SSS) within each line used for bisulfite-sequencing analysis. (**B**) Quantification of TAR in the three different F3 lines. Shown are relative abundances of leaves from 3 week-old plants over four distinct *Hpa* colonisation classes. For details, see Fig. 1B. Different letters above bars indicate statistical differences (Fisher’s exact, all-versus-all, FDR-adjusted; *p* ≤ 0.05). (**C**) Hierarchical clustering (Pearson correlation, Ward) of cytosine methylation profiles at CG, CHG and CHH sequence contexts. Lines are colour-coded to match the line annotations in panel A. Letters indicate replicate population samples within a line. Numbers at edges indicate AU (approximately unbiased) and BP (bootstrap probability) *p*-values (%), respectively. Confidence values are only shown for edges where AU or BP *p*-values are < 100.

Previously, we found that TAR can be transmitted over one stress-free generation into the F2 generation^8^. To investigate whether TAR and heritable *Pst*-induced changes in CG DNA methylation can be transmitted over two stress-free generations, we propagated the F2 lines from our previous study^8^ with one additional generation under similar (short-day) conditions. This resulted in one control line that had been exposed for at least three generations to stress-free conditions (MMM), one line that had been exposed to high levels of *Pst* stress in the first generation and kept free of biotic stress for two successive generations (SMM), and one line that had been exposed to high *Pst* stress over all three generations (SSS; Fig. 3A). Compared to the first F3 experiment, levels of disease stress in this second F3 experiment were higher, since plants had been inoculated at least five times per generation with *Pst*. Taking the MMM line as a control for TAR, we observed increased resistance in F3 progeny in both the SMM and the SSS lines (Fig. 3B). However, TAR in SMM progeny was statistically lower than TAR in SSS progeny (Fisher’s exact test, *p* < 0.05), suggesting that exposure to *Pst* over multiple generations results in enhanced TAR, or that the level of TAR decreases over stress-free generations.

**Figure 4:**
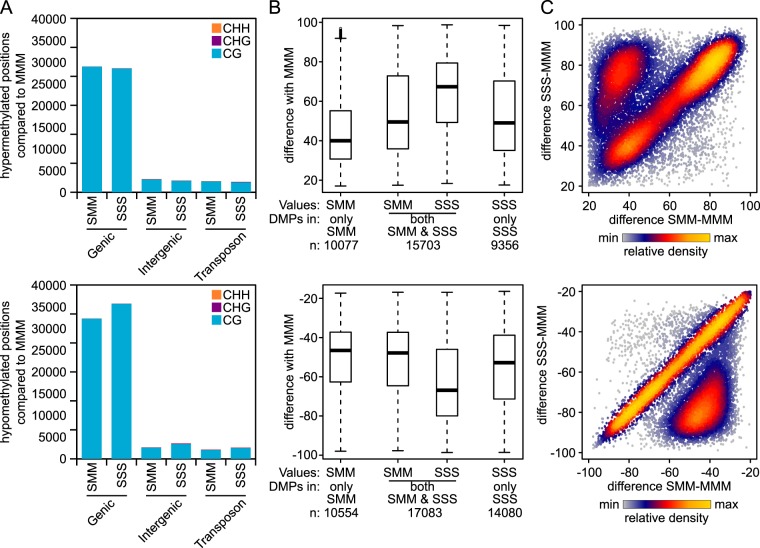
Genomic features and levels of hyper- and hypo-methylated DMPs in F3 progenies after one initial or three successive generations of disease stress. (**A**) Number of DMPs mapping to genomic features. For details, see Fig. 1D. Top panel: hyper-methylated DMPs; Bottom panel: hypo-methylated DMPs. (**B**) Levels of hyper- and hypo-methylation for DMPs in SMM and SSS progenies. Box plots show differences in percentage points between the MMM line and the SMM or SSS line. Shown are DMPs that are unique for the SMM line (left), unique for the SSS line (right), or shared between the SMM and SSS line (middle). Top panel: hyper-methylated DMPs; Bottom panel: hypo-methylated DMPs. (**C**) Comparison of the level of differential methylation for shared DMPs between SMM and SSS progenies. Correlation plots present the percentage point difference of the SSS line (y-axis) against that of the MMM line (x-axis). Top panel: hyper-methylated DMPs; Bottom panel: hypo-methylated DMPs. To aid interpretation, data points are colour-coded according to the relative density of data points. Each data point represents a single DMP (n = 15,703 and 17,083 for hyper- and hypo-methylated DMPs, respectively).

As for the previous experiments, triplicate samples from each line, each consisting of a pool of four similarly aged leaves from 10 plants, were subjected to whole-genome bisulfite sequencing. Consistent with the global methylation patterns of the first F3 experiment (Fig. 2C), Pearson correlation analysis and PCA of cytosine methylation showed relatively high similarity between replicate samples within lines at CG context, which was not evident at CHG and CHH contexts (Figs 3C and S1C). Moreover, the clustering patterns for CG methylation showed differences according to ancestral stress treatment, whereas such clustering was absent for CHG and CHH methylation (Figs 3C and S1C). The correlative distance between SMM and SSS lines was smaller than the distance between the MMM line and the SMM or SSS line (Fig. 3C). While this clustering pattern could be caused by a gradual loss of *Pst*-induced DMPs in the SMM line or an additive effect of multi-generation *Pst* exposure on DMPs in the SSS line, part of this variation may also be caused by spontaneously occurring variation in CG methylation over multiple generations. Previous studies of Arabidopsis mutation accumulation (MA) lines have shown that changes in cytosine methylation can occur spontaneously over generations in the absence of any introduced stimuli^29^. Like the TAR-related DMPs (Figs 1D, 2D and 4A), these labile positions occur predominantly in CG context^29^. Since MMM plants had been separated from SMM and SSS plants for three generations, whereas SMM and SSS plants had only been separated for two generations (Fig. 3A), part of the observed clustering in this experiment could be a reflection of spontaneously occurring changes in CG methylation over generations.

To differentiate between *Pst*-induced changes and spontaneously occurring changes in DNA methylation, subsequent statistical analyses focused on the quantitative differences in CG methylation between the three progenies (Figs 4A and S3; Tables S2-S3). As observed in the first F3 experiment (Fig. 2D), the majority of statistically significant DMPs in comparison to the MMM line occurred in CG context within genic regions (Fig. 4A). Furthermore, of all 35,136 hyper-methylated DMPs, 15,703 (44.7%) were statistically significant in both the SMM line and SSS line. The hypo-methylated DMPs revealed a similar level of overlap: of all 41,717 CG DMPs, 17,083 (40.9%) were statistically significant in both SMM and SSS (Fig. 4B). Notably, the level of hyper- or hypo-methylation within the group of shared DMPs was stronger in SSS plants than in SMM plants (Fig. 4B). As is further quantified in Fig. 4C, 3,269 hypo- and 4,981 hyper-methylated shared DMPs were more pronounced (≥20%points difference) in SSS plants, whereas only 201 hypo- and 201 hyper-methylated DMPs were more pronounced in SMM plants. These results indicate that the duration of disease exposure in previous generations has a dose-dependent impact on the level of differential CG DNA methylation in populations of F3 plants. Furthermore, this quantitative difference in CG methylation cannot solely be explained by spontaneously occurring variation in CG methylation over generations.

### The relationship between TAR-related CG methylation and spontaneously occurring variation in CG methylation

To examine the relationship between TAR and spontaneously occurring DNA methylation, we compared the ancestry of all F1 and F3 lines to the correlation structure of all CG positions from all lines and samples used in this study (Fig. S4). This analysis revealed a general resemblance between the overall pedigree structure and the correlation structure of the CG methylation tree. This indicates that the dominant variation in DNA methylation between the lines is determined by spontaneously occurring variation. However, *Pst*-exposed lines from the second F3 experiment, in which plants had been exposed to relatively high levels of disease stress (SSS and SMM), clustered widely apart from all other lines, including the corresponding control MMM line (Fig. S4). By contrast, samples from the MMM control line of this second F3 experiment clustered closer to samples from the first F3 experiment, where lines had been exposed to either stress-free conditions, or relatively moderate levels of disease stress. Since the MMM line had been separated for the same number of meiotic events (8) from all other F3 lines, this discrepancy between pedigree structure and CG correlation pattern suggests that high levels of disease stress alter the rate of CG epi-mutation between the F1 and F3 generation.

To further explore the relationship between TAR and spontaneously occurring variation in CG methylation, we compared the set of shared TAR-related DMPs from the second F3 experiment (Fig. 4B,C) to previously reported methylation-labile and methylation-stable positions from Arabidopsis MA lines (MA-DMPs and MA-NDMPs, respectively)^29^. The core set of TAR-related DMPs contained more labile MA-DMPs than stable MA-NDMPs (Fig. S5), reinforcing the notion that TAR preferentially acts on labile CG positions. Furthermore, using spontaneously occurring DMPs between the control lines in our first F3 experiment as a comparator (Mock 1 versus and Mock 2; Fig. 2), we detected a statistically significant shift in the distribution of hypo-methylated TAR-DMPs towards labile MA-DMPs, reinforcing the notion that TAR alters spontaneous epimutation rates at labile positions.

**Figure 5:**
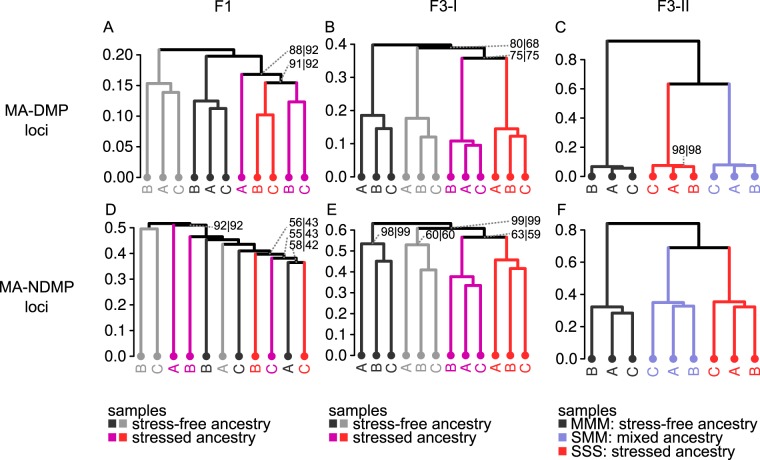
The relationship between TAR-related CG methylation and spontaneously occurring variation in CG methylation. Shown are correlation clusters of all experiments (Pearson correlation, Ward), using previously reported labile (MA-DMP; **A**–**C**) or stable (MA-NDMP; **D**–**F**) cytosine positons between mutation accumulation lines of Arabidopsis^29^. Numbers at edges indicate AU (approximately unbiased) and BP (bootstrap probability) *p*-values (%), respectively. Confidence values are only shown for edges where AU or BP *p*-values are <100.

To examine whether the global TAR-related changes in CG methylation are determined by methylation-labile positions only, or whether it also targets methylation-stable positions, we re-analysed CG correlations for all three experiments, using only labile MA-DMPs or stable MA-NDMPs from the MA lines^29^. Pearson correlation analysis of the labile MA-DMPs revealed clustering according to ancestral treatment for all experiments, although this pattern was statistically less robust in the F1 experiment and the first F3 experiment (AU *p*-value 0.88 and 0.75 respectively; Fig. 5A–C). Correlation analysis of the stable MA-NDMPs showed loss of clustering of F1 samples by treatment (Fig. 5D). Conversely, the clustering by treatment was still evident for samples of the first F3 experiment, which was of similar statistical robustness as the clustering of labile MA-NDMPs (AU *p*-value 0.63; Fig. 5E). Furthermore, the clustering of samples by treatment and line was still highly significant in the second F3 experiment (Fig. 5F). Thus, the TAR-related pattern of CG methylation in the F3 generation is not solely determined by methylation-labile positions. This indicates that the impact of ancestral disease stress on DNA methylation extends beyond methylation-labile CG positions only.

### TAR-related patterns of DNA methylation are not consistent across regions

Due to the spontaneous variation in methylation of single cytosines, it is possible that our positional analysis had relatively poor statistical discriminative power to detect TAR-related patterns of DNA methylation. To address this possibility, we examined whether regional patterns of DNA methylation yield better correlations with ancestral disease stress and TAR. To obtain a global clustering pattern of regional DNA methylation, methylation data across 100 bp windows of the genome were summarised and subjected to cluster analysis. This regional approach did not cluster replicate samples by ancestral treatment in any methylation context, with the exception of the lines from the second F3 experiment in CG context (Fig. S6). We then searched for differentially methylated regions (DMRs) between control and *Pst*-exposed lines. In line with the results of the global clustering, we found very low numbers (<10) of potential DMRs in the F1 and first F3 experiment. The exception was our comparison between lines from the second F3 experiment, where we identified 121 and 163 hyper- and hypo-methylated DMRs between SMM and SSS lines, respectively (Table S8). The overlap between this experiment and our first F3 experiment was a single hypo-methylated DMR in AT1G23400, which encodes a homologue of the maize chloroplast splicing factor CAF2. This gene plays no known role in plant defence, and it is also not induced during infection^35^. Overall, these results indicate that the TAR-related changes in DNA methylation are not concentrated within distinct regions. This is consistent with our finding that the global patterns of TAR-related DNA methylation in F3 lines occurs at GC positon in gene bodies (Figs 2D and 4A), which typically show more dispersed patterns of cytosine methylation in comparison to methylation of TEs^36^.

### Global impacts of TAR on CG methylation in F3 progeny are determined by a relatively small set of DMPs

**Figure 6:**
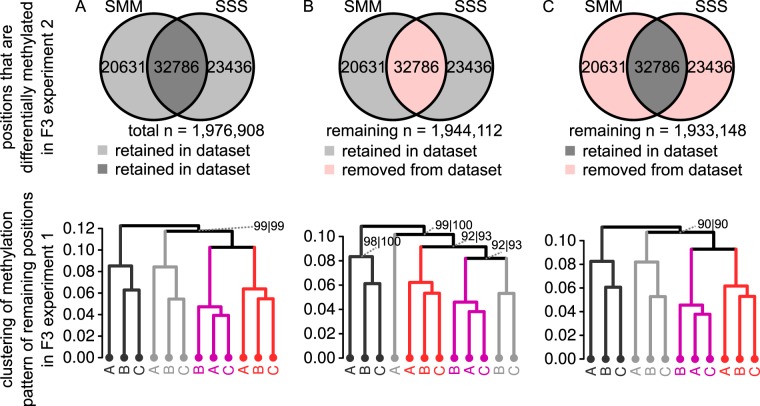
Contribution of a core set of CG DMPs to global patterns of TAR-related DNA methylation in F3 progeny. (**A**) Positive control. Hierarchical clustering (Pearson correlation) based on 1,976,908 CG positions from the dataset of the first F3 experiment. See legend to Fig. 2C for details. (**B**) Hierarchical clustering (Pearson correlation) based on 1,944,112 CG positions from the dataset of the first F3 experiment (Fig. 2) after removal of 32,786 shared CG DMPs between SMM and SSS from the second F3 experiment (Fig. 4C). (**C**) Negative control. Hierarchical clustering (Pearson correlation, ward method) based on 1,933,148 CG positions from the dataset of the first F3 experiment (Fig. 2) after removal of the 43,760 unshared DMPs between SSS and SMM from the second F3 experiment (SMM only + SSS only; Fig. 4C). For simplicity, a single Venn diagram is shown for hyper- and hypo-methylated positions combined. A DMP is considered shared if the difference with the mock is in the same direction in SMM and SSS. A small minority of DMPs (307; 0.4%) that are in opposite directions are considered as non-overlapping and counted in both non-overlapping sections.

The clustering patterns of positional CG methylation in both F3 experiments point towards a global impact of disease stress on gene body methylation. However, it remains unclear to what extent these global patterns are determined by a conserved set of stress-responsive DMPs. To address this, we examined the consistency of *Pst*-induced CG DMPs between the first and second F3 experiment, which were conducted under different environmental growth conditions. To this end, the 2,941 CG DMPs from the first F3 experiment with relatively low levels of applied disease pressure were compared to the 32,786 shared CG DMPs from the second F3 experiment, in which plants had been exposed to relatively high levels of disease pressure. Of the 1,509 hyper-methylated DMPs from the first experiment, only 231 were identical with the 15,703 hyper-methylated DMPs from the second F3 experiment (15.3% and 1.5% of the individual sets, respectively). Similarly, of the 1,432 hypo-methylated DMPs from the first F3 experiment, only 182 showed overlap with the 17,083 hypo-methylated CG DMPs from the second F3 experiment (12.7% and 1.1% of the individual sets, respectively). It thus appears that the majority of TAR-related CG DMPs in the F3 generation varies between independent lines and experiments. However, considering the dose-dependent relationship between ancestral disease stress and the level of differential methylation at selected positions (Fig. 4B,C), it is possible that this low overlap in DMPs between experiments is caused by differences in disease pressure between the first and second F3 experiment. Consequently, levels of differential methylation for many of the shared SSS-SMM DMPs in the second (stronger) F3 experiment may have been too weak and/or variable in the first (weaker) experiment to meet the statistical criteria, even though they may have had a contribution to global TAR-related clustering in this experiment (Fig. 2C). To test this hypothesis, we examined whether the 32,786 shared DMPs from the second F3 experiment contribute to global TAR-related clustering of CG methylation in the first F3 experiment (Fig. 6A). To this end, we removed the DMPs identified in our second F3 experiment (shared between SSS and SMM lines) from all CG positions in the first experiment, and re-clustered the remaining CG positions by Pearson correlation. The resulting correlation tree no longer grouped the *Pst*-inoculated F3 populations together (Fig. 6B), indicating that the set of shared CG positions from the second F3 experiment determines the global patterning of TAR-related methylation in the first F3 experiment. When the loci corresponding to the 43,760 unshared DMPs between SSS and SMM lines were removed from the analysis, the disruption of TAR-related clustering was substantially less pronounced (Fig. 6C). This *in silica* experiment suggests that a relatively small set of loci determines the global patterning of TAR-related CG methylation in F3 plants from independent experiments.

### Genes carrying TAR-related DMPs do not show defence-related gene ontology enrichment

To examine the cellular and biological functions of the genes carrying the set of 32,786 shared DMPs, we performed gene ontology (GO) term enrichment analysis. Comparison of genes with DMP-containing promoters against a genomic background of all genes revealed no statistically significant GO term enrichment (Tables S4 and S6). By contrast, comparing genes with DMP-containing gene bodies against a genomic background revealed statistically enriched GO terms, including protein phosphorylation (hyper-methylated DMPs) and plasma membrane-localised components (hypo-methylated DMPs; Table S5 and S6). However, when assessing GO term enrichment of genes containing the 44,067 un-shared DMPs, which do not correlate with TAR (Table S7), similar enrichment levels were found for most of these terms (Table S6).This suggests that genes with certain GO-annotated functions are more likely to carry DMPs, regardless of their correlation to the TAR phenotype. Two exceptions were the GO term ‘glycosyl transferases’ (GO:0016757) and the GO term ‘carbon-nitrogen ligase activity with glutamine as amino-N-donor’ (GO:0016884), which were more strongly enriched in the gene set with shared TAR-DMPs (Table S6). Notably, none of these GO terms are related to plant defence, suggesting that changes in gene body methylation do not have a direct contribution to plant defence. Interestingly, however, genes carrying the set of shared TAR-DMPs from the second F3 experiment that overlap with stable NDMPs from the MA lines showed noticeably stronger GO term enrichment than the corresponding control group (i.e. genes carrying spontaneously occurring DMPs that overlap with MA-NDMPs; Fig. S5). The majority of these terms are related to epigenetic activity, such as DNA (de)methylation and DNA repair.

## Discussion

Arabidopsis develops TAR after recurrent exposure to biotic stress, which is associated with priming of inducible defences and epigenetic mechanisms^8–10^. Since there is ample evidence that patterns of differential DNA methylation can be transmitted faithfully over multiple generations^8,9,12,13,15–19^, DNA methylation is the most plausible mechanism by which TAR is transmitted. This is further supported by previous evidence that mutations in DNA (de)methylation machinery affect TAR^13^. In the current study, we have examined global impacts of ancestral disease on DNA methylation in independent experiments. Our study provides four lines of evidence that ancestral disease influences heritable DNA methylation in Arabidopsis. Firstly, global cluster analysis of CG methylation in independent F3 progenies showed greater correlation between lines based on ancestral stress treatment (Figs 2C and 3C). Secondly, the intensity of the shared DMPs in SSS and SMM progenies from a second F3 experiment correlated with the level of ancestral disease exposure (Fig. 4C), suggesting a dose-dependent effect of ancestral biotic stress. Thirdly, the TAR-related pattern of CG methylation in the first F3 experiments was disrupted after removing the CG positions that showed differential methylation in both SMM and SSS progeny of the second F3 experiment (Fig. 5B). Finally, comparison of TAR-related DMPs with previously characterised CG positions from mutation accumulation lines of Arabidopsis revealed that ancestral stress predominantly acts on methylation-labile CG positions, but extends to methylation-stable CG positions. Together, these results suggest that the TAR-related pattern of CG methylation in the F3 generation involves a stress-inducible component that is not solely determined by stochastic variation in CG methylation and that is reproducible between independent experiments under varying growth and stress conditions.

Previous within-generation studies have shown that exposure to biotic stress changes DNA methylation at transposable elements (TEs) at both CG and non-CG context^13,19^. Although our analyses revealed similarly sized changes in DNA methylation, it did not detect statistically robust differences in non-CG methylation at TEs. In addition, it remains difficult to explain why we, and others^34^, failed to establish a correlation between phenotype and global DNA methylation patterns in F1 plants. Furthermore, the correlations between global DNA methylation patterns and TAR in F3 plants could only be detected in CG context, and the majority of differentially methylated CG sites occurred at gene bodies (Figs 1D, 2D and 4A). Although gene body methylation is common in Arabidopsis, where approximately one third of all genes display gene body methylation, its contribution to gene regulation and phenotype remains a matter of debate^27,37^. Whilst gene body methylation has been implicated in the regulation of gene transcription^38^, silencing of cryptic promoters^39^, and alternative gene splicing^40,41^, a recent study failed to identify a clear role of gene body methylation in the control of gene expression in Arabidopsis^42^. It has even been proposed that gene body methylation is a consequence of gene expression and/or previous epigenetic events, without having a direct regulatory impact on gene transcription^43^. Accordingly, it is possible that the global patterns of TAR-related CG methylation in F3 plants are an indirect consequence of ancestral disease stress, which do not directly contribute to TAR. This is supported by our GO term analysis of TAR-DMP-carrying genes, which failed to detect an enrichment of plant defence-related terms (Table S6). Thus, whilst the signature of ancestral disease stress on CG methylation may mark TAR in F3 plants, it does not necessarily contribute to the TAR phenotype itself.

If the global patterns of TAR-related CG methylation in F3 plants do not cause TAR, then what epigenetic mechanisms are responsible for TAR? Since mutations in DNA (de)methylation machinery deregulate TAR^13^, we propose that DNA methylation is still responsible for TAR, but that the causal changes in DNA methylation occur in regions that were insufficiently covered by our method of bisulphite sequencing. Due to the relatively short sequencing reads generated by bisulfite-sequencing (50–126 nt), its accuracy in detecting differential methylation in repetitive DNA regions, such as TEs, is limited. This would also explain why we failed to detect consistent TAR-related changes in regional DNA methylation (Fig. S5). We estimate that ~30% of annotated TEs in the reference genome could not be analysed reliably for methylation status, because sequence reads could not be mapped unambiguously in all samples. This low coverage was particularly pronounced at the (peri)centromeric regions (Fig. S7), which are highly active in terms of DNA methylation and heterochromatin formation^44,45^. Biotic stress has been shown to reduce methylation levels in these regions^46^, and changes in methylation in these regions have been reported to control complex plant traits^47,48^. Accordingly, we cannot exclude that our bisulfite sequencing analysis has missed relevant changes in (peri)centromeric DNA methylation, and that the observed changes in CG gene body methylation in the F3 generation reflect an indirect response to ancestral disease stress that develops over subsequent generations.

Gene body methylation at symmetrical CG context is largely maintained in a binary fashion: the cytosines are either methylated or un-methylated^37^. However, the TAR-related differences in CG methylation between SMM and SSS progenies were quantitative, and not binary. It is possible that TAR was not transferred to all individuals in the progeny. Considering that we quantified DNA methylation in pooled leaf samples from 10 plants, variation in the number of individuals expressing TAR could create quantitative differences at single positions. Secondly, the pattern of DNA methylation in leaf tissues of progeny will likely differ from that of the stem cells in the parental apical meristem, the gametes and the zygote, which could attenuate the binary nature of the differences. However, these mechanisms do not explain how quantitative differences in CG methylation are transmitted through the germline, and why SMM and SSS progenies in the F3 generation showed quantitative differences in TAR (Figs 3 and 4). Considering that our lines were propagated by single-seed descent, the binary nature of CG methylation predicts that SMM progeny should show a similar levels of CG methylation and TAR to either SSS or MMM progeny. Since this was not the case, we propose that both responses are regulated indirectly via quantitative mechanisms. Based on the hypothesis postulated above, we propose that multiple binary differences in pericentromeric DNA methylation quantitatively regulate TAR and DNA methylation at distant gene bodies. For instance, hypo-methylation of pericentromeric TEs could generate non-coding RNAs that *trans*-regulate chromatin structure and DNA methylation at distant loci^49^. Alternatively, changes in long-distance heterochromatic interactions with (peri)centormeric regions could quantitatively influence chromatin structure, DNA methylation and gene expression at distant loci^50,51^.

Elucidating the exact regulatory mechanisms of TAR will require further large-scale integrated analyses of DNA methylation and gene transcription at carefully selected time points after pathogen challenge. Moreover, to investigate potential *trans*-regulatory mechanisms controlling epigenetic responses to ancestral disease stress, these studies should include global analysis of small RNAs and heterochromatic genomic interactions by chromatin confirmation capture analysis^52,53^. Finally, the possibility that stress-induced changes in cytosine methylation influence the rate of DNA mutation^54^, for instance through accelerated rates of cytosine mutation^55^ or changes in DNA sequence caused by stress-activated TEs^56^, remains to be answered. To address this question, lines would need to be exposed to recurrent disease pressure over much longer timescales. Although time- and resource-consuming, this approach would enable elucidation of the complex interaction between environment, the epigenotype and the genotype over evolutionarily-relevant timescales, and generate insights that are of direct relevance to a range of disciplines, including (epi)genetics, plant evolution, and plant immunity.

## Methods

### Plant Material

Arabidopsis seeds were stratified in water at 4 °C in darkness for 3–5 days before sowing on a 4:1 soil:sand mixture (first F3 experiment) or on *Jiffy-7* peat pellets (Jiffy; F1 experiment and second F3 experiment). All lines used in this study were generated from seeds of a single individual descended from at least three stress-free generations. Plants were grown under 8.5 h (F1 experiment and second F3 experiment) or 16 h (first F3 experiment) light photoperiods at ~120 µmol s^−1^ m^−2^ light intensity, 21–22 °C and 80% relative humidity. Inoculations with *Pst* DC3000 were performed either five-six times within a generation (F1 experiment and second F3 experiment) as previously described^8^, or three times within a generation (first F3 experiment). The MMM, SMM and SSS lines studied in the second F3 experiment were produced from previously generated F2 lines^8^. Each new generation was started from seed collected from a single individual of the previous generation (Fig. S4). For each of the three experiments, plant material for DNA extraction was collected from healthy five-week-old plants that had been cultivated as described above. Each sample for bisulfite-sequencing consisted of four leaves collected from 10 plants, enabling the quantification of average levels of cytosine methylation within this population.

### Disease resistance assays

To test resistance against the downy mildew pathogen *Hyaloperonospora arabidopsidis* (*Hpa*), seedlings were grown for three weeks before spray-inoculation with a suspension of 10^5^ conidiospores ml^−1^ from isolate Waco9. Spores were harvested from hypersusceptible Ws-NahG seedlings on which a stock of the pathogen is maintained. Stocks were maintained by rinsing sporulating seedlings in water, filtering the resulting spore suspension through Miracloth (Merck Millipore) to remove debris, and spraying the suspension on fresh 2–3 week-old seedlings. After spray inoculation, plants were left to air-dry for 30–60 minutes and then kept at 100% humidity. For trypan blue staining, samples were collected in 100% ethanol and then transferred to a staining solution of 1 part lactophenol-trypan blue solution (0.067% w/v trypan blue, 33% w/v phenol, 33% v.v glycerol and 33% v.v DL-lactic acid in dH_2_O) and 2 parts 100% ethanol. Tubes containing samples were incubated in boiling water twice for 1 minute with a 5 minutes interval at room temperature, and then left at room temperature to incubate for 3–5 hours. Samples were stored in 60% w/v chloral hydrate at least overnight before *Hpa* colonisation was scored. Typically, 150–250 leaves from a total of 30–50 plants per line were assigned to different colonisation classes; class I: no hyphal colonisation; class II: hyphal colonisation but no sporulation; class III: hyphal colonisation with conidiophores; class IV: extensive hyphal colonisation with conidiophores and oospores.

### DNA Extraction and bisulfite sequencing

Samples were snap-frozen in liquid nitrogen upon collection and stored at −80 °C until extraction of DNA, which was performed with the GenElute Plant Genomic DNA Miniprep Kit (Sigma; F1 Experiment and first F3 experiment) or a CTAB protocol (second F3 experiment). For CTAB DNA extraction, frozen samples were treated with 1 ml of CTAB buffer (2% CTAB, 100 mM Tris-HCl pH 8, 1.4 M NaCl, 20 mM EDTA, 1% PVP- 40; 2 µl ml^−1^ 2-Mercaptoethanol was added immediately before use), homogenised and incubated for 60 minutes at 65 °C. One volume of chloroform was added and mixed by vortex before centrifuging 8 minutes at 9500 ***g***. In a clean tube, DNA was precipitated from the aqueous phase for 30 minutes at room temperature using one volume of isopropanol. After centrifuging for 15 minutes (16,500 ***g*** at 4 °C), the pellet was washed with 70% ethanol and centrifuged for 5 minutes (16,500 ***g*** at 4 °C). The pellets were air-dried and resuspended in water. RNA was removed by precipitation with 2 M of LiCl, incubation at 4 °C overnight and centrifugation for 20 minutes (16,500 ***g*** at 4 °C). DNA was precipitated with 2.5 volumes of absolute ethanol for 4 hours at −20 °C and centrifuging for 20 minutes (16,500 ***g*** at 4 °C). DNA pellets were washed with 70% ethanol and centrifuged for 5 minutes (16,500 ***g*** at 4 °C), air-dried and re-suspended in water. DNA from all samples were analysed for integrity and quantity by electrophoresis (0.8% agarose gels containing 5 μg/mL ethidium bromide) and normalised to 200 ng μl^−1^. Samples were bisulfite-treated and Illumina-sequenced by Zymo (paired-end, 50 bp reads; second F3 experiment) or GATC (paired-end, 126 bp reads; F1 experiment and first F3 experiment).

### Methylation calling

Sequencing data was trimmed using trimmomatic^57^ (‘HEADCROP:9 CROP:101 SLIDINGWINDOW:4:24’ or ‘HEADCROP:5 CROP:46 SLIDINGWINDOW:4:24’ for GATC sequences and Zymo sequences, respectively) and filtered to retain only sequences longer than 36 nt. Sequences were then aligned in single-end and paired-end mode through bismark^58^ (version 0.15.0), using bowtie2^59^ (version 2.2.8). Picard tools (version 2.17.11; http://broadinstitute.github.io/picard) were then used to merge single reads that did not align in paired-end mode or whose mate was missing after filtering with the paired-end alignments. Sequencing and alignment statistics are provided in Table S8. Output files were sorted using sambamba (version 0.6.0) and then read into methylKit^60^ (version 0.9.5) in R (version 3.2.4; https://www.R-project.org/) using the function read.bismark (mincov = 3, minqual = 20). Reads aligning to the plastids were separated from the information for the nuclear chromosomes. Bisulfite conversion efficiency was determined from the chloroplast sequences, as the chloroplast DNA is not normally methylated. Conversion rates were estimated at 99.37% − 99.60% for all samples (Table S8). A single replicate from one of the *Pst*-treated lines in the F1 experiment was discarded from further analyses as both its sequencing coverage patterns and methylome pattern differed significantly from all other samples. Data was filtered to remove extremely high coverage regions (filterByCoverage; hi.perc = 99.9), normalised (normalizeCoverage) and then united, keeping only positions for which at least two replicates per line had sufficient coverage (unite; min.per.group = 2 L). In CG context counts for both strands were merged (unite; destrand = TRUE), whereas in all other contexts this options was set to false. To enable fair comparisons between all lines, the data from the experiments were united into a single table. Differences in cytosine methylation were called between different treatments for each individual experiment, using a dispersion shrinkage for sequencing data (DSS) method to correct for over-dispersion^61^. DMPs were defined as differences that were statistically significant at 5% FDR. Differentially methylated regions (DMRs) were called using DSS and a relaxed per position *p*-value < 0.01 as cut-off, together with a delta value of 0.1. DMRs were filtered to retain regions with a minimum overall methylation difference of 10%.

### Data analysis and feature annotation

Statistically significant changes in distribution of *Hpa* colonisation classes were determined by Fisher’s exact tests. Hierarchical clustering was performed in R, using methylKit’s clusterSamples function with default settings (including filtering low variation (sd < Q50) sites), with bootstrapping performed using pvclust^62^ (nboot = 10,000). PCAs were performed with methylKit’s PCASamples function. Heatmaps were generated using gplots^63^ and scatter plots using heatscatter from R package LSD^64^. The TAIR10 genome annotation (www.arabidopsis.org) was used. Bedtools was used to create a general feature annotation, where an annotation of transposon was given priority over annotation of a gene, and a gene annotation was prioritised over an intergenic region annotation. MA-DMP and MA-N-DMP tables were kindly provided by Dr. Claude Becker and classed according to occurrence of DMP and/or N-DMPs; in rare cases where the one cytosine at CG context was classed as DMP and the palindromic cytosine as N-DMP, the DMP annotation was chosen. PlantGSEA^65^ and GOrilla^66^ were used to study enrichment of GO terms.

## Electronic supplementary material

Supplementary Figures S1–7

Supplementary Tables S1–9

## Data Availability

All sequencing data has been deposited at the European Nucleotide Archive (ENA) under accession number PRJEB20931.
