# Supplementary material for: The relationship between transgenerational acquired resistance and global DNA methylation in Arabidopsis
Source: Sci Rep. 2018 Oct 3;8:14761. doi: 10.1038/s41598-018-32448-5 (PMC6170496; doi:10.1038/s41598-018-32448-5)
Supplement: Supplementary file 1 — Supplementary Figures S1–7 [file 41598_2018_32448_MOESM1_ESM.pdf]

Supplementary Figures

The relationship between transgenerational acquired resistance and global DNA methylation in Arabidopsis

Joost H.M. Stassen[1*], Ana López Sánchez[1], Ritushree Jain, David Pascual Pardo, Estrella Luna, Lisa M. Smith and Jurriaan Ton*.

Department of Animal and Plant Sciences, Faculty of Science and P3 Centre for Translational Plant Science, Western Bank, University of Sheffield, Sheffield S10 2TN, United Kingdom.

[1] Equal contribution
* Corresponding authors: j.stassen@sheffield.ac.uk and j.ton@sheffield.ac.uk

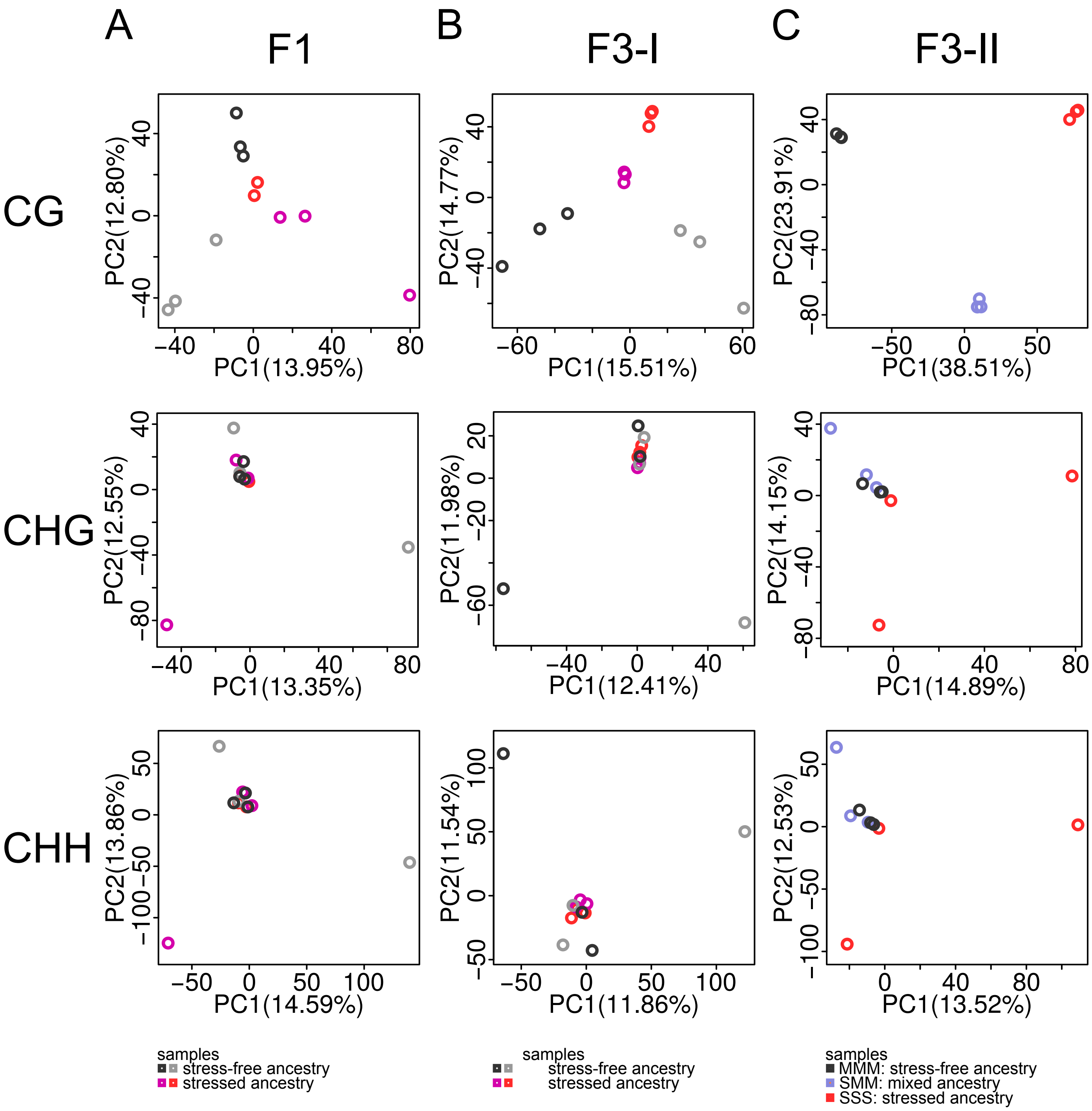

Supplementary Figure 1: Principle component analysis (PCA) of CG, CHG and CHH methylation patterns in all each experiment.

Colours indicate different lines, corresponding to the colour codes shown in the main figures.

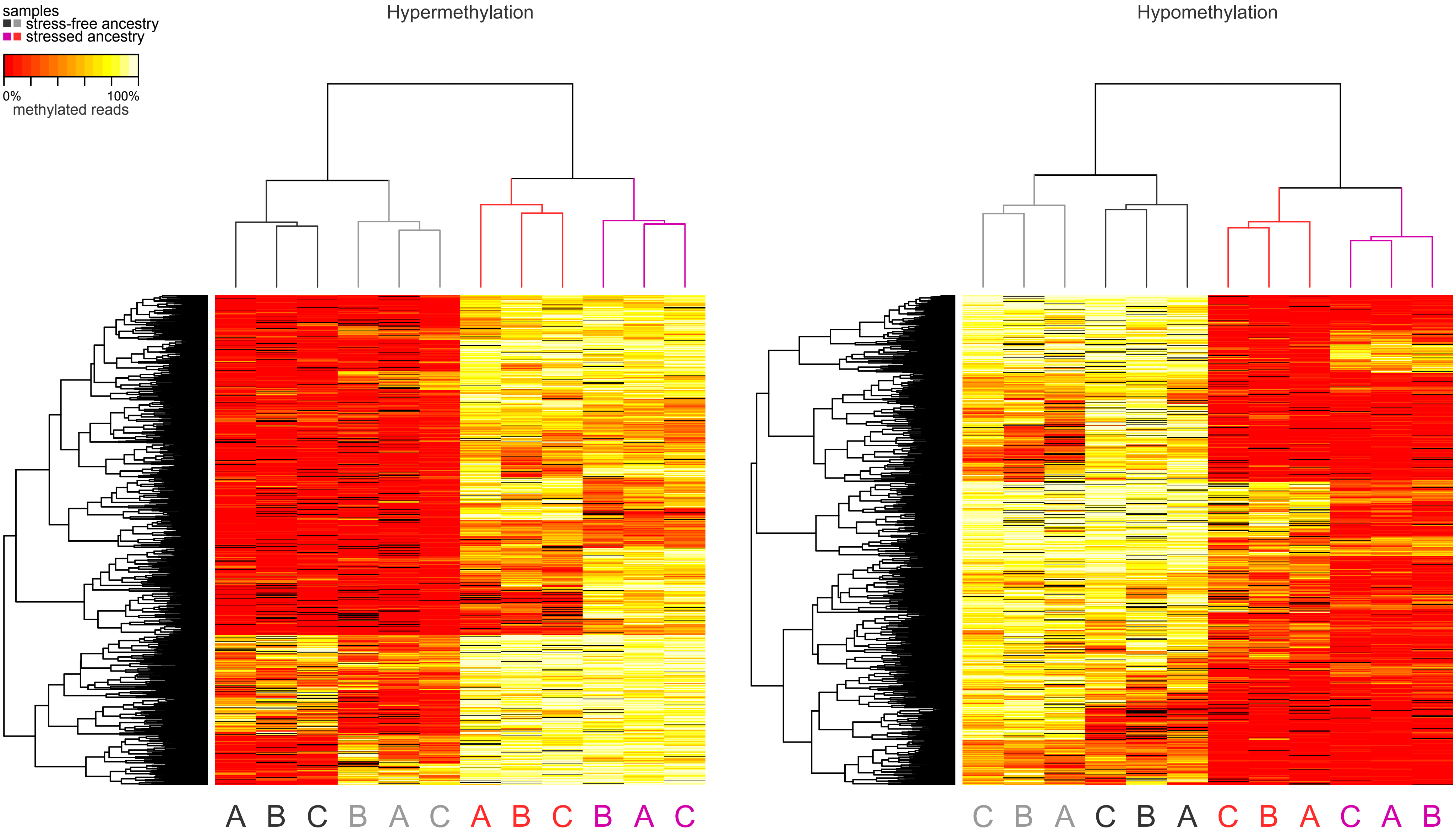

Supplementary Figure 2: Methylation levels at differentially methylated CG positions between *Pst*- and mock-treated F3 progenies (first F3 experiment). Heatmap values represent percentages of methylated reads at 2,941 CG positions that are differently methylated in *Pst*-exposed lines relative to mock-treated control lines of the first F3 experiment (1,509 hyper-methylated, 1,432 hypo-methylated). Values were clustered by DMP and sample (Pearson correlation, Ward). Letters at the bottom indicate replicate population samples within lines, whose colours match the progeny annotations in Fig. 2A.

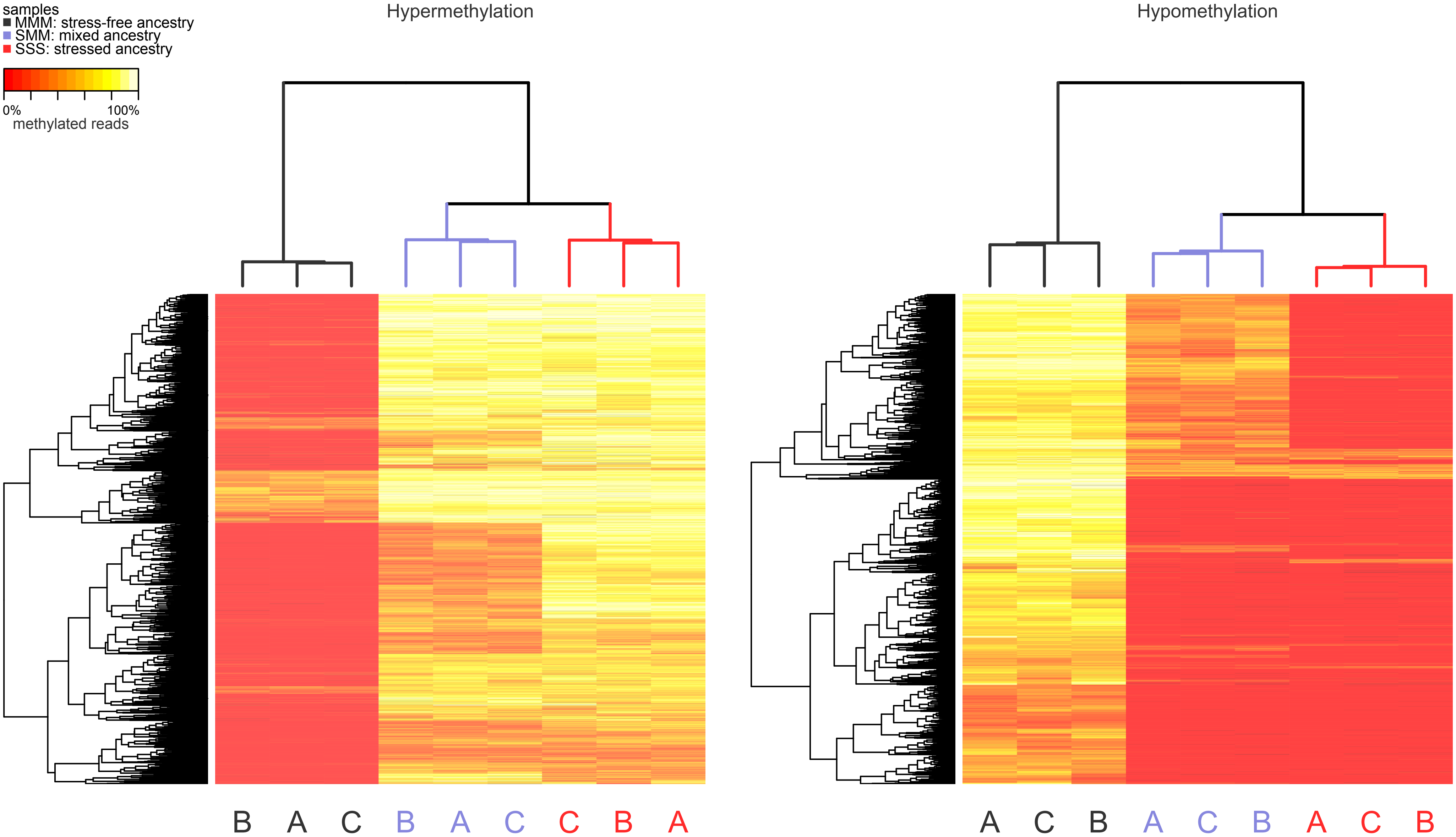

Supplementary Figure 3: Methylation levels of differentially methylated CG positions in SMM and SSS plants relative to MMM plants (second F3 experiment). Heatmap values represent percentages of methylated reads at 32,786 CG positions that are differently methylated in both the SMM and SSS lines relative to MMM plants. Values were clustered by DMP and sample (Pearson correlation, Ward). Letters at the bottom indicate replicate population samples within lines, whose colours match the progeny annotations in Fig. 3A.

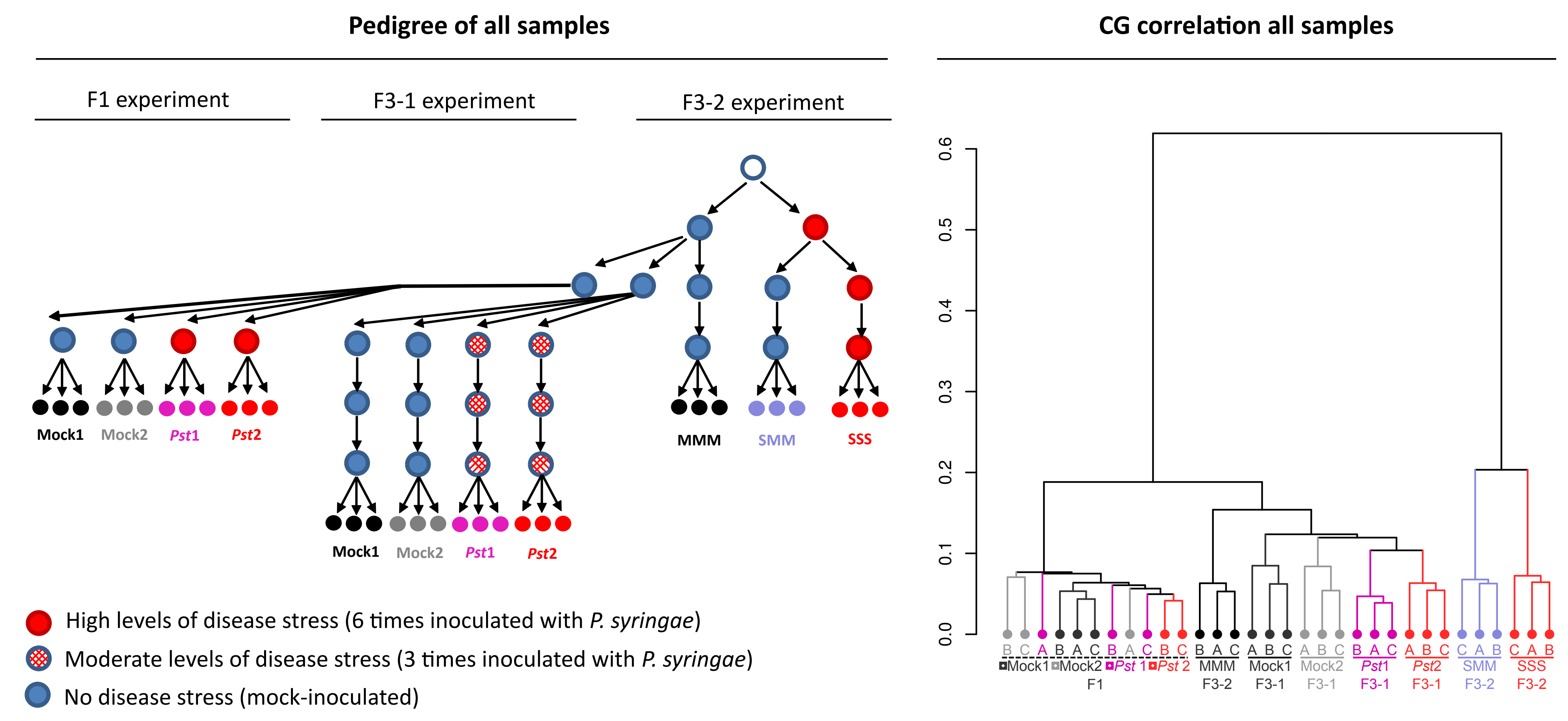

Supplementary Figure 4: Comparison between pedigree structure of all lines (left) and correlation clusters of CG methylation in all samples (right). Clustering of CG methylation was based on Pearson correlation (Ward method). For details, see main figures.

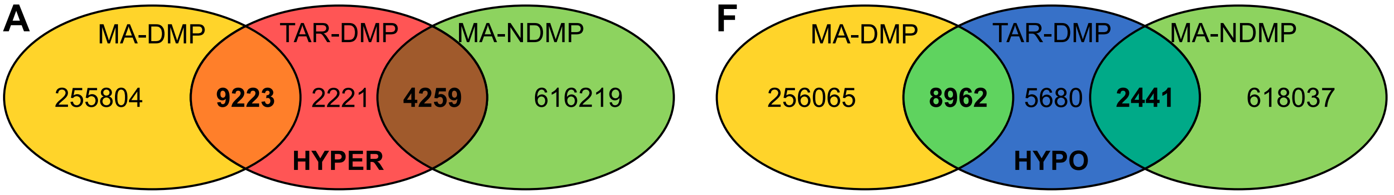

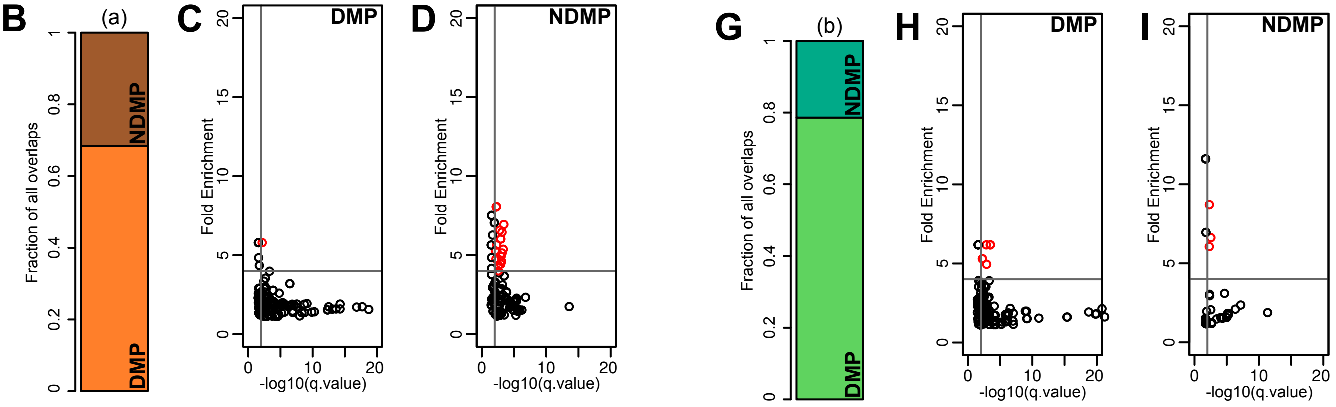

E

GO terms overlap TAR-DMP with MADMPs	F.E.	q.value
GO:0098813 nuclear chromosome segregation	5.79	6.91E-03

GO terms overlap TAR-DMP with MA-NDMPs	F.E.	q.value
GO:0048194 Golgi vesicle budding	6.94	3.77E-04
GO:0044728 DNA methylation or demethylation	5.37	4.70E-04
GO:0006900 vesicle budding from membrane	6.45	7.28E-04
GO:0016050 vesicle organization	5.13	7.14E-04
GO:0006405 RNA export from nucleus	4.6	8.58E-04
GO:0006304 DNA modification	4.91	1.03E-03
GO:0051168 nuclear export	4.43	1.16E-03
GO:0006306 DNA methylation	6.02	1.17E-03
GO:0006305 DNA alkylation	6.02	1.14E-03
GO:0080188 RNA-directed DNA methylation	6.58	1.68E-03
GO:0016458 gene silencing	4	2.71E-03
GO:0006891 intra-Golgi vesicle-mediated transport	4.34	2.68E-03
GO:0007030 Golgi organization	4.34	2.63E-03
GO:0032386 regulation of intracellular transport	5.64	4.88E-03
GO:0006342 chromatin silencing	8.06	5.67E-03
GO:0045814 negative regulation of gene expression, epigenetic	8.06	5.59E-03
GO:0045332 phospholipid translocation	8.06	5.52E-03
GO:0048507 meristem development	8.06	5.44E-03
GO:1902275 regulation of chromatin organization	4.75	6.35E-03
GO:0060968 regulation of gene silencing	5.27	7.79E-03

J

GO terms overlap TAR-DMP with MA-DMPs	F.E	q.value
GO:0006342 chromatin silencing	6.18	3.38E-04
GO:0045814 negative regulation of gene expression, epigenetic	6.18	3.28E-04
GO:0070988 demethylation	4.95	1.24E-03
GO:0080111 DNA demethylation	6.18	1.38E-03
GO:0035510 DNA dealkylation	5.3	5.73E-03
GO:0048507 meristem development	5.3	5.66E-03

GO terms overlap TAR-DMP with MA-NDMPs	F.E.	q.value
GO:0044728 DNA methylation or demethylation	6.63	2.90E-03
GO:0006289 nucleotide-excision repair	8.71	4.98E-03
GO:0006304 DNA modification	6.06	5.10E-03

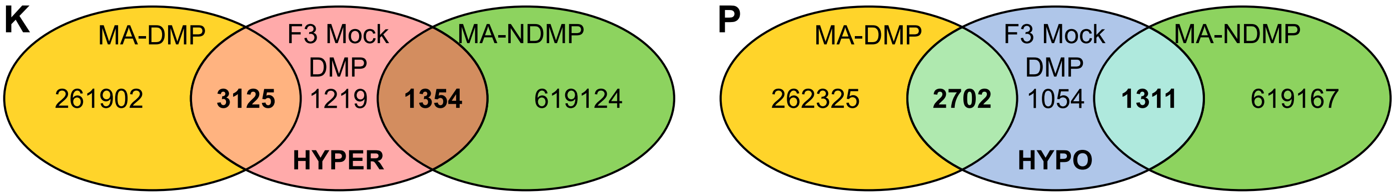

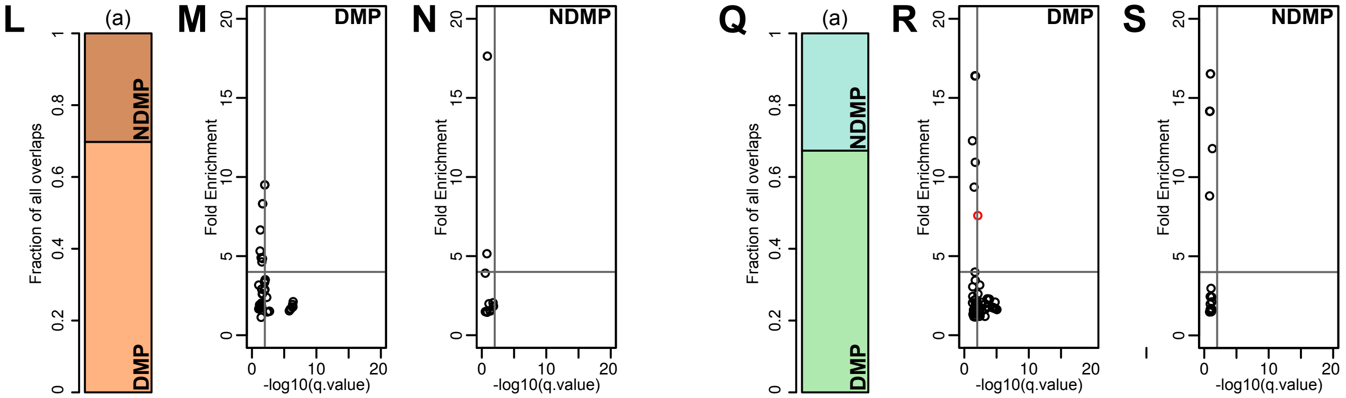

O No GO term enrichment found

T

GO terms overlap Mock-DMPs with MA-NDMPs	F.E.	q.value
GO:0070646 protein modification by small protein removal	7.56	8.25E-03

Supplementary Figure 5: Overlap between DMPs from the second F3 experiment and previously reported labile (MA-DMP) or stable (MA-NDMP) cytosine positons between mutation accumulation lines [29].
A-E: overlap with hyper-methylated TAR-DMPs, **F-J**: overlap with hypo-methylated TAR-DMPs, **K-O**: overlap with spontaneous hyper-methylated DMPs between mock-inoculated lines (F3 Mock-DMPs), **P-T**: overlap with spontaneous hypo-methylated DMPs between mock-inoculated lines (F3 Mock-DMPs). Venn diagrams show numbers of positions overlapping with MA-DMPs or MA-NDMPs (**A,F,K,P**). Stacked bar graphs show relative distributions of DMPs between MA-DMPs and MA-NDMPs (**B,G,L,Q**). Different letters above the stacked bars indicate statistically different distributions ($\chi 2$, p value < 1e-10). **C,D,H,I,M,N,R,S**: Enrichment of gene ontology (GO) terms of genes carrying TAR- or Mock-DMPs that overlap with MA-DMP or MA-NDMPs (*p* value < 1e-3). Graphs plot enrichment value against statistical significance. Terms with ≥ 4-fold enrichment and q-value < 0.01 are shown in red and are further specified in panels **E,J,O,T**. F.E.: fold enrichment.

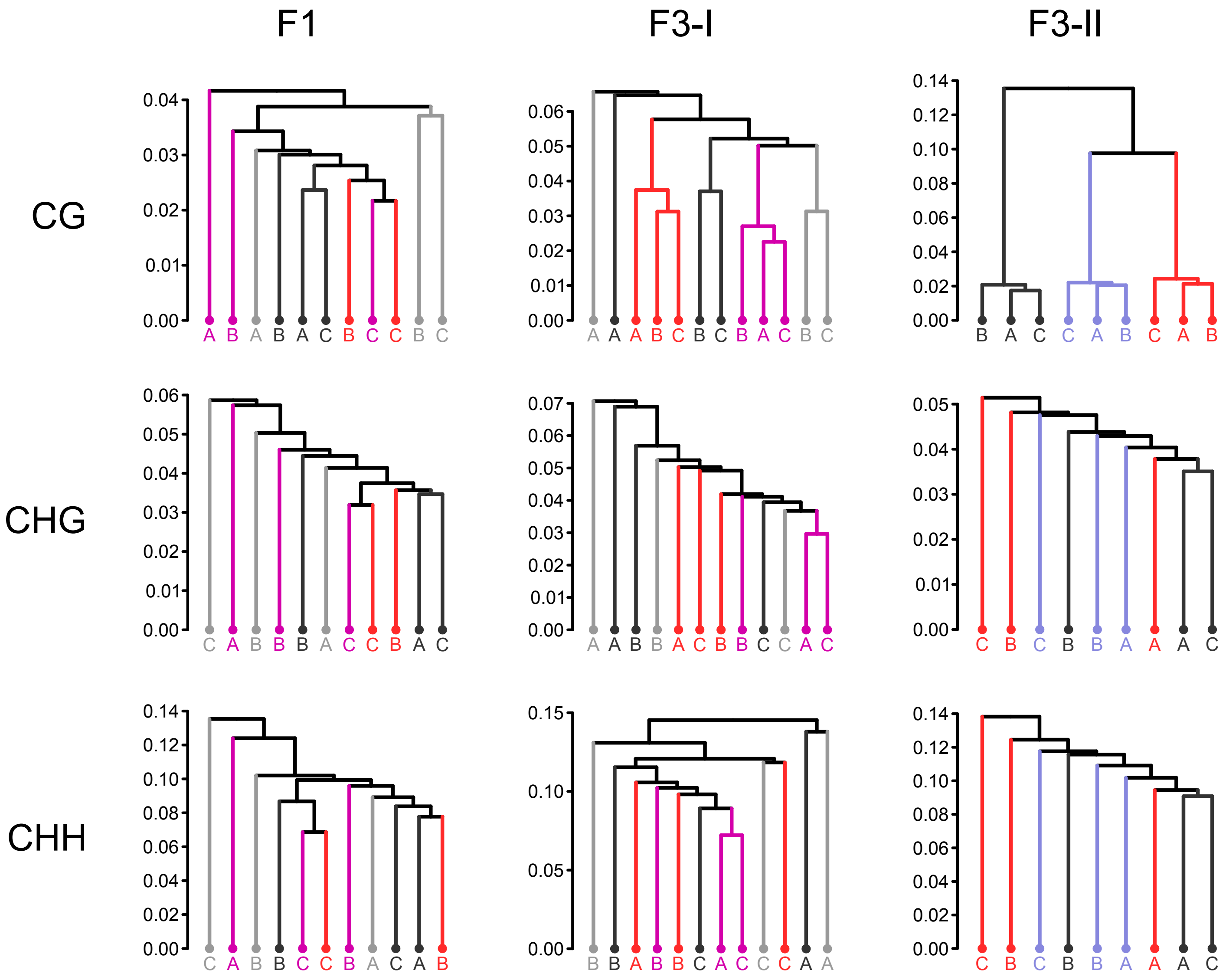

Supplementary Figure 6: Hierarchical clustering (Pearson correlation, Ward) of regional profiles of cytosine methylation.
Regional correlations were based on 100-bp-window averages (step size 50 bp) at CG, CHG and CHH sequence contexts for all lines in each of the experiments. Lines are colour-coded to match the line annotations in the corresponding pedigrees, included in the main Figures. Letters indicate replicate population samples within a line.

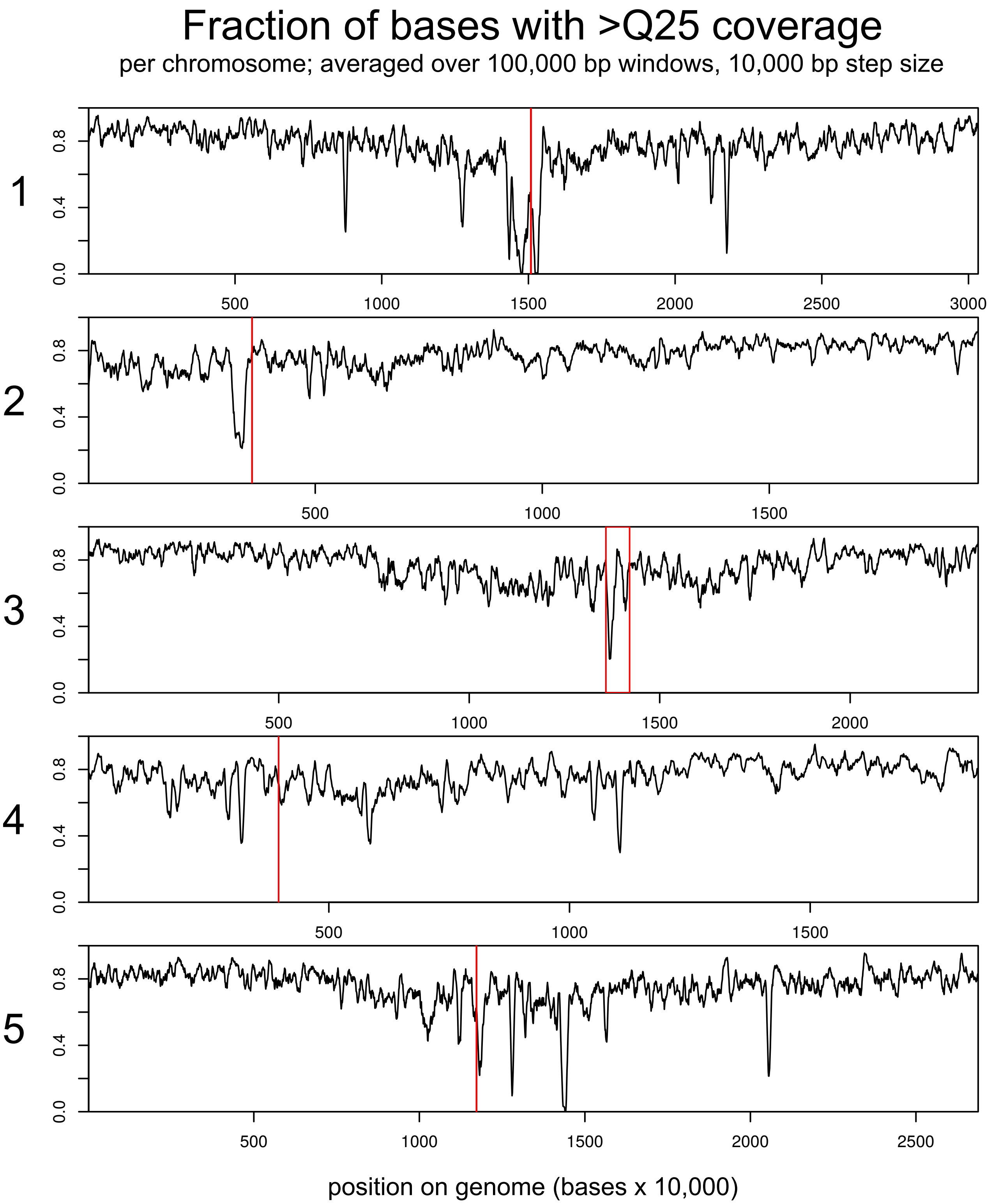

Supplementary Figure 7: Global coverage of bisulfite-sequencing reads for all five chromosomes of Arabidopsis.

Shown are fractions of bases with greater than first quartile coverage from the first replicate sample of the MMM line (second F3 experiment). Fractions are calculated over a 100,000 base pair region, with a step size of 10,000 base pairs. Red lines/boxes indicate centromere annotations.
